# Physical Activity Comparison Between Body Sides in Hemiparetic Patients Using Wearable Motion Sensors in Free-Living and Therapy: A Case Series

**DOI:** 10.3389/fbioe.2018.00136

**Published:** 2018-10-17

**Authors:** Adrian Derungs, Corina Schuster-Amft, Oliver Amft

**Affiliations:** ^1^Lehrstuhl für Digital Health, Friedrich-Alexander Universität Erlangen-Nürnberg, Erlangen, Germany; ^2^Research Department, Reha Rheinfelden, Rheinfelden, Switzerland; ^3^Institute for Rehabilitation and Performance Technology, Bern University of Applied Sciences, Burgdorf, Switzerland; ^4^Department of Sport, Exercise and Health, University of Basel, Basel, Switzerland

**Keywords:** IMU, acceleration, stroke, rehabilitation, metabolic equivalent, effect size

## Abstract

**Background:** Physical activity (PA) is essential in stroke rehabilitation of hemiparetic patients to avoid health risks, and moderate to vigorous PA could promote patients' recovery. However, PA assessments are limited to clinical environments. Little is known about PA in unguided free-living. Wearable sensors could reveal patients' PA during rehabilitation, and day-long long-term measurements over several weeks might reveal recovery trends of affected and less-affected body sides.

**Methods:** We investigated PA in an observation study during outpatient rehabilitation in a day-care center. PA of affected and less-affected body sides, including upper and lower limbs were derived using wearable motion sensors. In this analysis we focused on PA during free-living and clinician guided therapies, and investigated differences between body-sides. Linear regressions were used to estimate metabolic equivalents for each limb at comparable scale. Non-parametric statistics were derived to quantify PA differences between body sides.

**Results:** We analyzed 102 full-day movement data recordings from eleven hemiparetic patients during individual rehabilitation periods up to 79 days. The comparison between free-living and clinician guided therapy showed on average 16.1 % higher PA in the affected arm during therapy and 5.3 % higher PA in the affected leg during therapy. Average differences between free-living and therapy in the less-affected side were below 4.5 %.

**Conclusion:** We analyzed PA of patients with a hemiparesis in two distinct rehabilitation settings, including free-living and clinician guided therapies over several weeks and compared MET values of affected and less-affected body sides. In particular, we investigated PA using individual regression models for each limb. We demonstrated that wearable motion sensors provide insights in patient's PA during rehabilitation. Although, no clear PA trends were found, our analysis showed patients' tendency to sedentary behavior, confirming previous lab study results. Our PA analysis approach could be used beyond clinical rehabilitation to devise personalized patient and limb-specific exercise recommendations in future remote rehabilitation.

## 1. Introduction

Physical activity (PA) is essential to maximize rehabilitation outcome of patients after stroke. Continuous sensor-based PA measurements could help to evaluate the recovery process, devise exercise adaptations, and investigate PA trends. Research suggested that PA of moderate and high intensity promotes stroke recovery, contribute to increased health and well-being, and reduces the risk of follow-up strokes (Gordon et al., 2004; Baert et al., 2012; Moore et al., 2013). Verschuren et al. (2015) and Paul et al. (2016) showed that stroke patients tend to sedentary behavior with light PA reducing rehabilitation efficiency and hence delay recovery. However, little is known about the gradual recovery process over weeks, months, and even years and how potential recovery trends could be evaluated in rehabilitation without specific tests and assessments. Assessing PA of patients after stroke in free-living is challenging, even in controlled rehabilitation settings, e.g., hospitals and clinics, activity patterns remain unclear (Field et al., 2013; Lacroix et al., 2016). So far, PA was primarily evaluated based on observation and subjective self-reports, rendering PA assessments unreliable (Billinger et al., 2014). In contrast, longitudinal sensor-based measurement could be used for PA quantification, support devising rehabilitation strategies and exercise recommendations, and facilitate recovery trend investigation, thus provide valuable insights in patients' recovery process. Moreover, PA differences between the affected and less-affected body side of upper and lower body extremities across multi-week rehabilitation period have not been investigated. Comparing body sides and body parts could reveal activity patterns and behavior related to functional limitations and movement compensation mechanisms. Therefore, analysing all extremities separately could facilitate targeted therapy to enhance motor functionality. We aim to utilize wearable sensors and linear regression to estimate MET equivalents, which could lead to PA analysis beyond clinical rehabilitation settings.

PA is typically expressed in units of energy or activity counts, however, these units are not comparable. In contrast, metabolic equivalents (MET) quantify PA intensity on a relevant and comparable scale (Jette et al., 1990). To separate intensity levels, distinct thresholds were used. For example, Garber et al. separated very light (≤ 2.0 MET), light (≤ 2.0–3.9 MET) and moderate-to-vigorous (≥ 3.0 MET) intensities for healthy people (Garber et al., 2011). MET measurements were usually derived using a mobile spirometer and a close-fitting face-mask (Verschuren et al., 2016). However, spirometry might be suited for lab-controlled, short-term measurements only, e.g., treadmill-walking. Wearable sensors were increasingly used for practical PA measurement (Plasqui et al., 2005; Parkka et al., 2007; Kozey et al., 2010; Rand and Eng, 2012). used a thigh-worn sensor to assess sedentary behavior based on posture and PA measurements in a realistic rehabilitation setting. Fortune et al. validated Shimmer sensor-based MET estimation with an indirect calorimetry reference system in rheumatic patients, yielding a correlation of 0.82 (Fortune et al., 2011). Mortazavi et al. demonstrated that sensors placed closest to the primary location of movement resulted in the most accurate MET estimation during soccer exergaming in healthy participants (Mortazavi et al., 2013). However, activity patterns considering individual extremities in patients after stroke were not investigated. In this work, we used an established regression-based approach to estimate MET equivalents.

In stroke rehabilitation, where recovery can be a gradual process over weeks, months or even years, binary test decisions of significance might be insufficient to capture subtle changes over time, e.g., PA differences between the affected and less-affected side (Sullivan and Feinn, 2012). Hence, the measure of effect size (Hentschke and Stüttgen, 2011) could be used to quantify subtle differences between body sides on a continuous scale.

Although advantages of wearable sensors and their potential in healthcare have been found, few studies quantified the outpatients' PA to demonstrate free-living viability of sensor-based measurement (Dobkin and Dorsch, 2011; Iosa et al., 2012). In this work, we measured the outpatients' PA in day-long recordings in a day-care center. The patient-specific rehabilitation programmes and exercises were designed by therapists and not modified for this study. Our analyses provide insight into patients' PA, which could be used to personalize PA investigation in future remote free-living monitoring. In particular, this paper provides the following contributions:

1. We analyzed PA in free-living and therapy and investigated differences in affected and less-affected body sides of the upper an lower body using body-worn motion sensors to obtain insights in patient behavior during an out-patient rehabilitation process.
2. In an evaluation study, we recorded a total of 102 recording days over several weeks from eleven outpatients after stroke or brain tumor extraction to evaluate PA differences on a interpretable scale using regression-estimated MET equivalents. In addition, we compared average PA differences using two non-parametric statistical tests, determining significance and effect size, respectively.
3. We discuss clinical implications and limitations and illustrate how wearable sensors could be used in free-living to reveal activity patterns and evaluate recovery trends.

## 2. Methods

We describe an observation study including participants, study design, sensor data recording, and expected observations. We subsequently describe the PA calculation, the MET estimation, and statistical methods used.

### 2.1. Evaluation study

#### 2.1.1. Participants

Eleven patients with a hemiparesis were included in our observation study (5 females, aged 34–75 years, 56 ± 13 years, 4 wheelchair users, 48–335 days post-stroke), Table 1 contains detailed patient information. Patients were excluded if presenting additional motor function impairments caused by other/additional neurological diseases. Study participants visited routinely the rehabilitation clinics' day-care center at Reha Rheinfelden, Switzerland during a study period of up to 79 days. All study participants signed a written consent form before data recordings began. The Swiss Ethics committee of the canton Aargau, Switzerland approved the study (Application number: 2013/009). As part of the standard rehabilitation programme, each patient was routinely assessed by clinicians at the begin and shortly before the discharge, using the Extended Barthel Index (EBI). The EBI is a clinical assessment to estimate the patients' level of independence in accomplishing daily activities. Due to the longitudinal rehabilitation process where patients focused on the training of daily activities to improve independence, we expected increasing EBI scores. The EBI consists of 16 categories including mobility (walking and stairs), transfers (e.g., from bed to a chair and back), feeding, dressing, and similar. Scores from zero (patients need full support) to four (patients do not require support) were used to assess each category. Table 1 includes the average EBI scores for the complete assessment and the subcategory walking. The average EBI score for the complete assessment at the begin of the standard rehabilitation programme was 55.5 ± 6.3, the average EBI score at the end of the rehabilitation was 59.2 ± 4.6. The subcategory walking showed on average an increase of 0.5 scores.

**Table 1:** Patient information.

**ID**	**Cause of impairment**	**Locomotion [type]**	**Gender**	**Affected [side]**	**Age [years]**	**Rehab [days]**	**Rec. [days]**	**DAS [days]**	EBI^*^compl	**EBI Δ_compl_**	EBI^*^walk	**EBI Δ_walk_**
1	Stroke	Wheelchair	M	Left	57	79	11	335	51	+8	1	+1
2	Stroke	Walk	M	Right	47	18	8	135	63	+1	4	0
3	Stroke	Wheelchair	M	Right	53	77	10	164	51	+10	0	0
4	Stroke	Walk	F	Left	52	16	7	295	60	+1	4	0
5	Stroke	Walk	F	Left	74	35	10	134	50	+7	2	+1
6	Stroke	Walk	M	Left	38	66	11	90	63	+1	3	+1
7	Stroke	Wheelchair	M	Right	64	28	9	164	56	+3	0	0
8	Brain tumor	Walk	M	Left	34	28	11	84	64	0	4	0
9	Stroke	Walk	F	Left	72	30	7	116	48	+5	2	+2
10	Brain tumor	Wheelchair	F	Left	68	30	9	274	48	+9	0	0
11	Brain tumor	Walk	F	Left	55	28	9	152	57	0	4	0
Mean					56.3	39.5	9.3	176.6	55.5	3.6	2.2	0.5
SD					13.1	23	1.5	85.3	6.3	4.0	1.7	0.7

*Locomotion describes the patients' mobility (walker or wheelchair user), Rehab is the rehabilitation duration, Rec. is the number of study recording days, DAS are the days after stroke or brain tumor extraction (duration between stroke event or brain tumor extraction and rehabilitation entry). EBI scores at rehabilitation entry are denoted as, EBI^⋆^ compl for complete assessment; EBI^⋆^ walk for subcategory walking. EBI score differences between the first and last assessment are denoted as, Δ_compl_ for complete assessment; Δ_walk_ for subcategory walking*.

#### 2.1.2. Study design

All patients received a personalized therapy schedule according to their expected needs, considering the level of independence and health state. Patients followed daily routines and trainings according their therapy schedules, but had free time too. The day-care center focused on re-integration into free-living, where outpatients train activities of daily living (ADL) to promote independence after the rehabilitation, e.g., during socializing, laying tables and kitchen work, cleaning, computer work, leisure, resting, and similar. In addition, patients received individualized clinician-guided therapies, e.g., physio-, ergo- and water-therapy. In this study, patients were accompanied and observed by study examiners for 2–3 times per week. The study examiners followed the patients for up to eight hours per recording day and annotated observed patient activities using Android smartphones and the open-source CRNTC+ framework. For the study, an annotation catalogue including a total of 51 activity primitives was specified. Activity primitives included *walking, walking up/downstairs, sitting, arm and leg flexion/extension, arm and leg rotation, writing, reading, using phone, drinking*, and similar, to describe patient activities, clinician guided therapy exercises and unguided training exercises in the gym (Derungs et al., 2018a). During the recordings, including free-living and clinician guided therapies as well as gym training and vibration sessions, the study examiners marked starting and ending of activities using the smartphone-based annotation tool. Subsequently, data annotations were approved after data post-processing by two study examiners. In addition, we specified typical activity routines, such as eating/leisure, cognitive training, medical fitness, kitchen work, motor training, and resting as reference for potential subsequent behavior description. The study time spread over 1–3 months per patient (39.5 days on average, 79 days maximum), included 9.3 recording days on average per patient, and a total of 102 recording days. During free-living, patients performed various ADL without guidance. Therapy included clinician guided exercises, which were devised according patients' needs and daily health conditions. For example, if patients felt unwell, had pain or expressed discomfort, therapy was adapted by the clinician, e.g., including relaxation exercises to ease the patient's pain. Patients' therapy schedules, activities, and incidents were documented daily in case reports according clinical guidelines, including strolls, sports, resting phases, or when patients felt unwell.

The distinction between free-living and clinician guided therapy was motivated by the primary rehabilitation goal, i.e., to gradually transfer from acute treatment to outpatient rehabilitation and finally to independent free-living without clinician guided therapy. During the rehabilitation process, patients were only supervised by clinicians during physio- and ergo-therapy, contrary to free-living when patients followed ADL without guidance. The outpatient rehabilitation at the day-care center promoted ADL in free-living in a controlled environment. Beside free-living, clinician guided therapies were inherent part of the regular rehabilitation strategy, supporting patients in targeted re-learning of functional motor tasks, e.g., grasping, reaching, standing up, and sitting down. Hence, the standard rehabilitation settings free-living and clinician guided therapy were considered for our observation study to reveal PA insights.

Although patients were encouraged to exercise in the gym, the unguided training was not mandatory, hence patients' training commitment was not guaranteed. Similarly, vibration training was not part of the standard rehabilitation programme. Further, we considered follow-up fitness and vibration training after the clinical rehabilitation unlikely. Few times sensors were detached during the rehabilitation, i.e., during water therapy and lymph drainage massages. As a consequence, we excluded the non-standard gym training, vibration sessions, and when sensors were detached for this PA analysis, and summarized these activities as *other activities (excluded)*.

#### 2.1.3. Expected observations and measurements

So far, little is known about PA in free-living (Buma et al., 2013; Waddell et al., 2017). Hence, this study was designed to obtain insights in patients' PA which could be used to personalize exercise recommendations for follow-up training in free-living. We expected higher PA in the affected arm during clinician guided therapy than in free-living, as the regular training focused on improving functional limitations on the affected body side. In contrast to therapy, higher PA was expected in the less-affected arm during free-living, as patients tend to use the unimpaired side to overcome functional limitations (Lang et al., 2007; Michielsen et al., 2012). In addition, we expected smaller differences between lower extremities in free-living and therapy compared to the upper body, because restoring upper body functionality was prioritized in this study. Moreover, bi-pedal locomotion, sitting, and resting activities were expected to influence affected and less-affected leg equally, resulting in higher similarity compared to the upper body. The EBI sub-score for walking, see Table 1, further suggested that improvements in motor functions were expected in the upper extremities, as differences in the subcategory walking were on average 0.5 scores between begin and end of the rehabilitation. In general, we hypothesized that patients become more active during the rehabilitation, in particular during the clinician guided therapy. We further expected that increasing PA could lead to improvements in ADL and independent living, related to an average EBI score improvement of 3.6 scores. Therefore, we analyzed PA and recovery trends using sequentially distributed boxplots.

#### 2.1.4. Sensors and data recording

**Figure 1:**
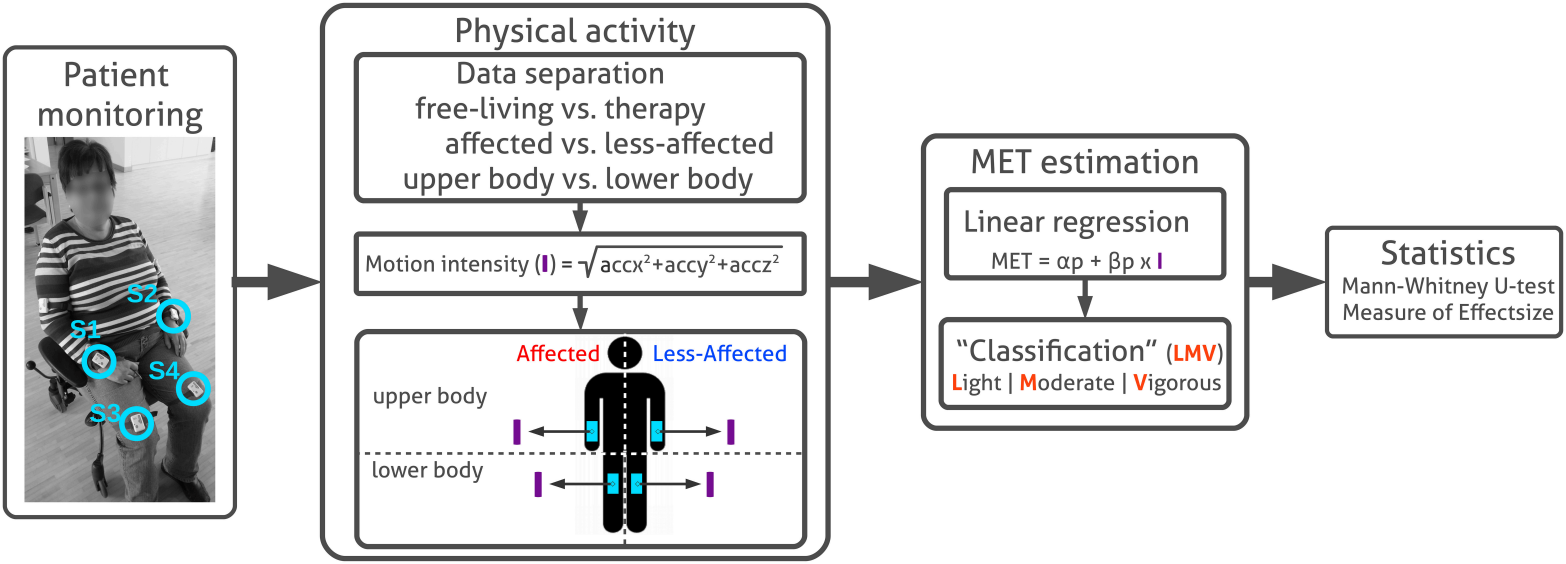
Patient monitoring and PA analysis. Sensor placement at writs and thighs are highlighted (S1, S2, S3, and S4). Acceleration sensor data are pre-processed to separate motion data derived in free-living and therapy using annotated routine labels. Subsequently motion intensity (I) is derived for each extremity. MET equivalents are estimated from motion intensity using linear regression models to divide intensity levels in categories light, moderate, and vigorous according distinct thresholds. In this work, we used the MET conversion to assess PA intensity on a relevant and comparable scale. We were interested in comparing PA between extremities, rather than estimating absolute MET equivalents. Finally, average PA differences between body sides in free-living and therapy were evaluated using non-parametric statistics, including the Mann–Whitney *U*-test and the *Measure of effect size*.

Motion data were recorded using Shimmer3 inertial measurement units (IMU) sensors (L x W x H = 51 × 34 × 14 *mm*^3^), consisting of 3-axis accelerometer, 3-axis gyroscope, and 3-axis magnetometer. The IMUs were configured to sample and log accelerometer (±4 g), gyroscope (±1,000 dps) and magnetometer (±1 Ga) data with 50 Hz to the sensors' internal SD-card. To calibrate the IMUs for these sensor ranges and to obtain optimal accuracy, we followed the Shimmer 9DoF calibration guidelines^1^. Each IMU was calibrated separately using the Shimmer calibration stand and corresponding calibration parameters were stored on the IMU and used for subsequent data processing. When patients arrived in the morning at the clinic, we attached in total six IMUs to both wrists, upper arms and thighs, using Velcro straps. Sensors were only temporary removed for water therapy or lymph drainage massages, and finally detached at the end of the day. Sensors were regularly checked by the study examiners during recordings to avoid variation in sensor orientation or position. Patients were not asked to perform sensor orientation and position checks. For the PA analysis described in this work only acceleration data derived from wrist- and thigh-worn IMUs were considered. Sensor positions are illustrated in Figure 1.

### 2.2. Physical activity analysis

We detail our PA analysis and motion intensity estimation, describe the MET estimation approach and subsequent statistical analysis as illustrated in Figure 1. MATLAB^2^ and the MES toolbox (Hentschke and Stüttgen, 2011) were used.

#### 2.2.1. Motion intensity calculation

Motion data from both wrist and thigh-worn IMUs were time-synchronized and merged according to their time stamps. For subsequent PA analysis, wrist-worn sensors were denoted as *upper* and thigh-worn sensors as *lower*. Sensor data from left and right body side were re-labeled with *Aff* (affected, impaired side) and *NonAff* (less-affected, healthy side). To separate free-living and therapy, and excluding fitness training in the gym and vibration training, we used annotated activities and routines to derive PA. We filtered the x-, y-, and z-acceleration using a high-pass filter with a cut-off frequency of 0.7 Hz similar to Ohkawara et al. (2011). Subsequent, motion intensity (**I**) was derived according to Equation (2):

(1) $$\text{I}=\sqrt{{\text{acc}}_{\text{x}}{}^{2}+{\text{acc}}_{\text{y}}{}^{2}+{\text{acc}}_{\text{z}}{}^{2}}$$

Motion intensity **I** was derived according to Zhang et al. (2012). For further data processing, we used *g*-values, thus divided the motion intensity by the gravitational force using the reference value 9.80 $\frac{m}{s^{2}}$^3^. Motion intensity calculations according to Equation (2), using 3-axis accelerometer data were described in published literature using different terminology, e.g., synthetic acceleration (Tsukahara et al., 2016), magnitude of acceleration (Ohkawara et al., 2011; Mortazavi et al., 2013), or norm (Zihajehzadeh and Park, 2016).

#### 2.2.2. Met estimation

**Figure 2:**
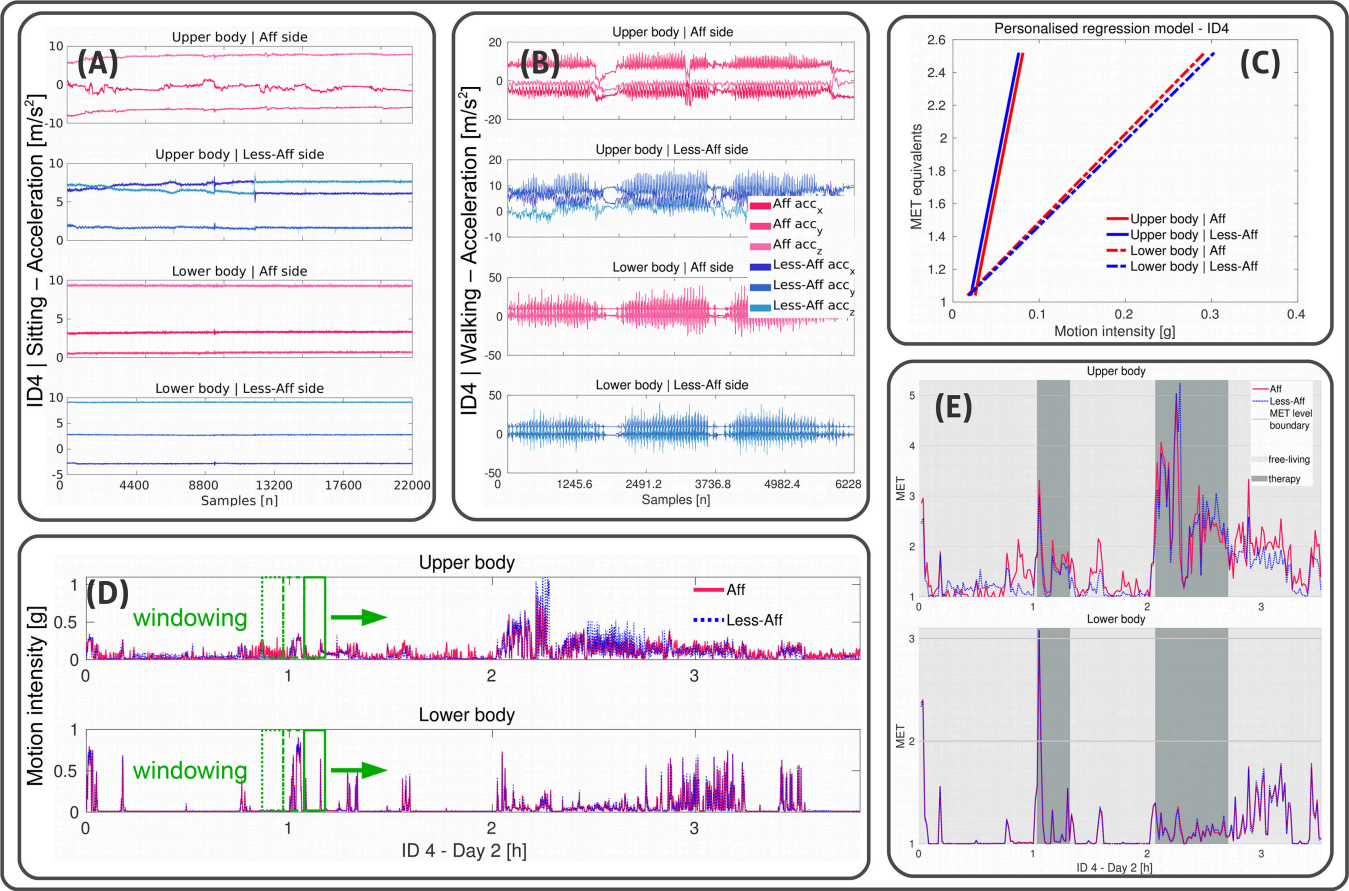
Illustration of the personalized MET estimation using linear regressions. Example data shown from recording day two of patient ID4 (walker). **(A,B)** Illustrate x-, y-, and z-acceleration data derived from wrist- and thigh-worn sensors of affected and less-affected body sides, and upper and lower body, respectively during sitting and walking. **(C)** Visualizes the regression model for each limb. Acceleration data in $\frac{m}{s^{2}}$ were further processed to derive the motion intensity in *g*-values. Reference MET equivalents were used to derive α and β using linear regression. **(D)** Shows the motion intensity for each limb derived during a complete recording day including free-living and clinician guided therapy. A non-overlapping windowing extracts data of 1 min epochs to estimate MET equivalents. **(E)** Illustrates estimated MET equivalents for each limb. Free-living and clinician guided therapy settings are highlighted for the upper and lower body affected and less-affected side.

In this work we used MET equivalents as an interpretable scale to quantify PA for each extremity independent of specific activities or therapy exercises. It is clear that MET equivalents estimated from one extremity includes bias. However, compared to an arbitrary energy unit or activity counts, we considered MET equivalents as interpretable. Moreover, instead of evaluating absolute MET equivalents, we were interested in comparing PA differences between the affected and less-affected body sides during free-living and therapy. MET equivalents were estimated using motion intensity derived from both wrist and thigh-worn sensors. We applied individual regression models for each limb to account for functional limitation and compensation mechanisms influencing affected and less-affected sides movement differently. Figure 2 illustrates our MET estimation approach.

Personalized regression models were first built, using the annotated activities *sitting* and *walking*, derived from each patient. In an iterative process over all recording days we extracted the day with the first sitting an walking instance. In case of several instances per day, we selected the sitting and walking instance with the longest duration. Subsequently, we derived the mean motion intensity of these instances which we used as baseline to determine the regression parameters α and β. Similar to Ohkawara et al. (2011), we used a linear regression model to estimate MET equivalents based on motion intensity derived from acceleration data according to the Equation (3):

(2) $$\text{MET}=\alpha_{\text{p}}+\beta_{\text{p}}\times \text{I}$$

where p refers to individual wrist- or thigh-worn sensors at affected and less-affected body sides, α and β denote offset and slope of the linear regression, respectively.

To derive the linear regression models' parameters α and β for the baseline, we used reference MET equivalents for sitting (1.04 MET) and walking (2.52 MET). The reference MET equivalents were based on mobile gas-analyser measurements derived from patients after stroke in a lab-controlled study (Verschuren et al., 2016).

Subsequently, a non-overlapping sliding windowing of 1 min was applied to derive MET estimates per limb during free-living and clinician guided therapy. Our pre-investigation showed that a non-overlapping windowing of 1 min provide sufficient information about the MET equivalents' data distribution for statistical PA analysis between body sides and rehabilitation settings. Moreover, different windowing lengths were used for PA analysis, ranging from 10 s for real-time measurements (Ohkawara et al., 2011) to 1 min for activity routine analysis (Crouter et al., 2006).

MET estimation in free-living is challenging, especially in longitudinal recordings over several months. Further, MET estimation based on activity type requires activity classification for subsequent regression parameter selection. Even in a clinical environment, activity classification is difficult due to patients functional limitations and variances in motions execution, as well as patient-individual therapy programmes. Hence, for this PA analysis we estimated MET equivalents without the need of classifying free-living activities and therapy exercises. Instead, we used a single regression approach, which was considered suitable for this PA analysis and could be used in further remote monitoring settings.

For the subsequent PA analysis, we separated activity levels into light, denoted as **L** (PA ≤ 2.0 MET), moderate, denoted as **M** (PA ≤ 2.0–3.0 MET) and vigorous, denoted as **V** (PA ≥ 3.0 MET) (Garber et al., 2011). We used linear regression equations (Crouter et al., 2006; van Hees et al., 2009) to estimate MET equivalents based on acceleration data only, hence a power-efficient implementation in resource-limited wearable systems are feasible. Moreover, power-demanding sensors, such as gyroscopes or computation-intensive algorithms could be avoided. Even if IMUs are implemented, switching of sensors like gyroscopes substantially reduces system energy consumption.

#### 2.2.3. Statistical analysis

PA differences between body sides and body parts were analyzed using the non-parametric Mann–Whitney *U*-test (Wilcoxon Rank-sum test) (Vickers, 2005) with a 5 % significance level (α = 0.05) and Cohen's U3 *Measure of effect size* (MESU3) (Hentschke and Stüttgen, 2011) to account for non-normal data distributions. The Rank-sum test evaluates the null hypothesis (**H**_0_) that MET equivalents of affected and less-affected body sides have equal medians, against the alternative hypothesis (**H**_1_) that they have not, using an arbitrary α-value. The Rank-sum test results in rejection (h = 1) or acceptance (h = 0) of **H**_0_. In contrast, MESU3 evaluates the magnitude of effects between two distributions on a continuous scale ranging from 0 to 1. Therefore, the continuous scale could reveal subtle changes during the gradual recovery process.

## 3. Results

### 3.1. Physical activity overview

**Figure 3:**
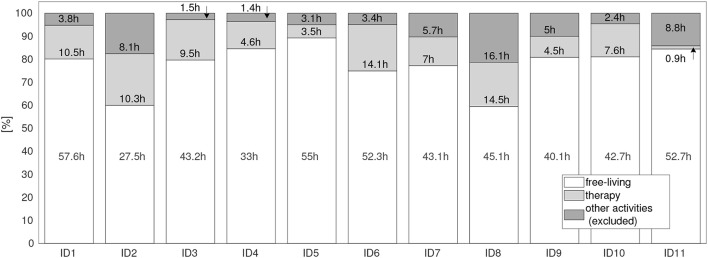
Motion data distribution during the complete rehabilitation period for all patients (ID1 to ID11). Settings: free-living (when patients performed everyday activities), therapy (when patients attended clinician guided therapies), and other activities. Other activities were excluded from the analysis, covering times for irregular training, e.g., vibration sessions, fitness training in the gym, as well as times where the sensors had been detached.

The PA distribution derived from sensor data during the rehabilitation period is summarized in Figure 3 for each patient. In total, 638.6 h of sensor data were derived from all patients, including 492.3 h (77.1 %) of free-living and 86.9 h (13.6%) recorded during therapy. In total 59.3 h (8.9 %) of recording were not considered in the analysis, of which 40.3 h (6.3 %) were recorded in gym sessions, 2.5 h (0.4 %) in vibration training, and 16.6 h (2.6 %) when sensor were detached. Gym and vibration training were not part of the standard rehabilitation programme, hence excluded. For example, only four vibration sessions for ID1 and ID7, three for ID3, two for ID2, ID4, ID6, ID8, and ID11 and no sessions for ID5, ID9, and ID10 occurred during all recordings. On average, the vibration sessions accounted for 1.2 % (44 min) for patients ID1, ID3, and ID7; 0.49 % (13.5 min) for ID2, and less than 0.032 % (1 min) for ID4, ID6, ID8, and ID11 for the complete rehabilitation duration. Gym training accounted between 1.6 % (52 min) for ID3 and 13.7 % (8.5 h) for ID11 for the complete rehabilitation duration.

**Figure 4:**
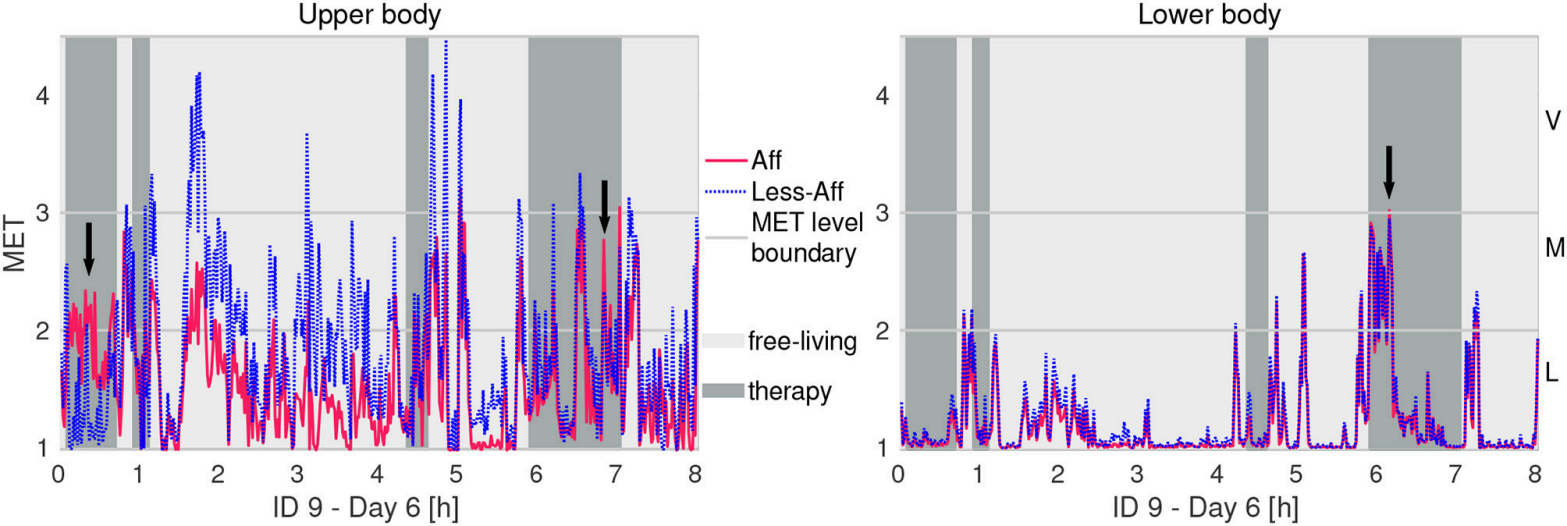
Example of physical activity in MET equivalents of recording day 6 of patient ID9 (walker). Letters **L**, **M**, and **V** denote thresholds for light, moderate, and vigorous activity intensity, respectively. Arrows indicate higher activity intensity in the affected side compared to the less-affected side.

Figure 4 illustrates exemplarily the PA during a recording day for the walking patient ID9 using MET equivalents. We observed mostly higher intensity in the less-affected upper body compared to the affected side. Further, ID9 showed mostly light activity below 2 MET equivalents in the lower body. Visual inspection of both PA time-series graphs suggested high similarity between body sides.

**Figure 5:**
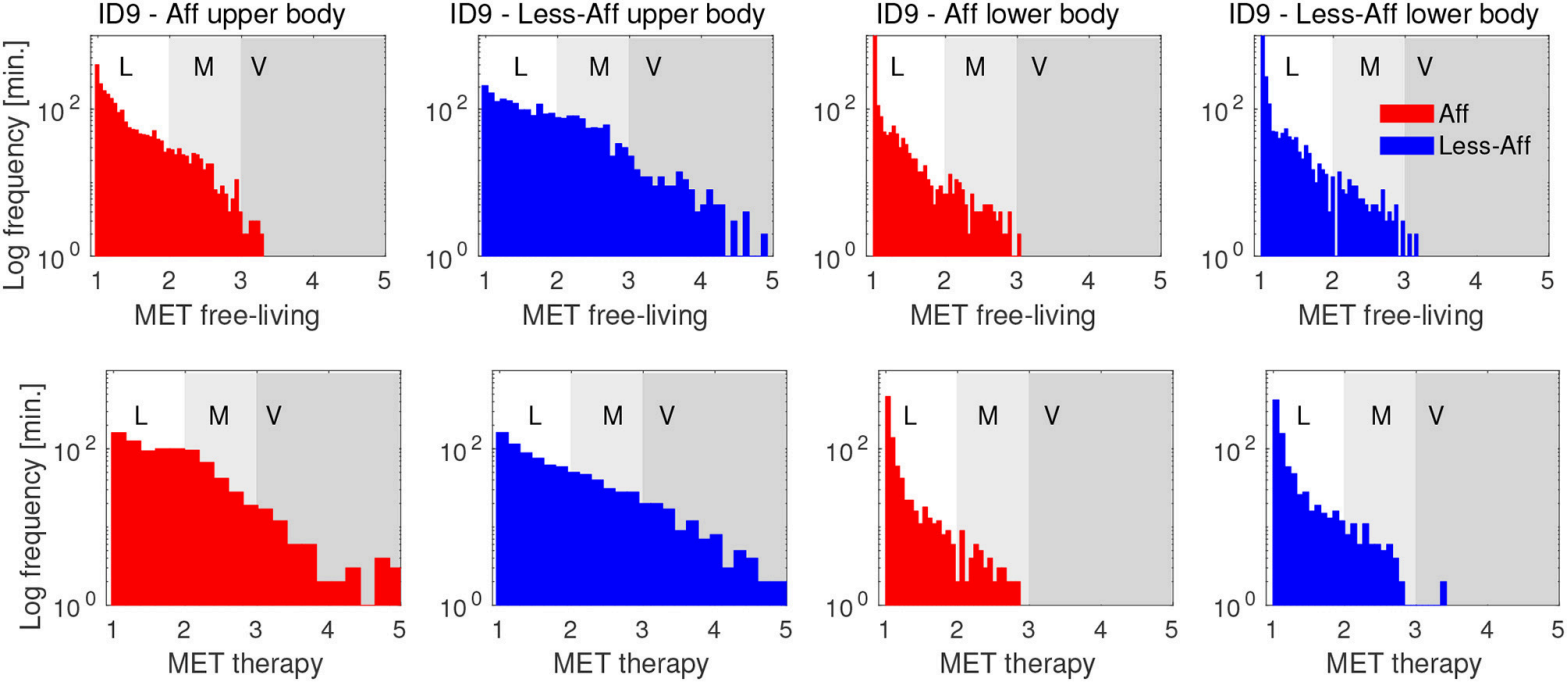
Histograms of PA in MET equivalents for affected and less-affected upper and lower body for patient ID9. The distribution show PA derived from free-living and therapy during the complete rehabilitation period of patient ID9. PA distributions show a positive skew, indicating mostly light intensity, corresponding to sedentary behavior. The y-axis is log-scaled to emphasize small values in the distribution. Letters **L**, **M**, and **V** denote thresholds for light, moderate, and vigorous activity intensity, respectively.

Histograms in Figure 5 show the PA distribution during the complete rehabilitation process of ID9 exemplarily. During free-living the patient's affected hand showed mostly light to moderate activity, whereas the less-affected side included segments of vigorous intensity. During therapy, we observed increased activity of the affected side up to vigorous intensity level. During therapy, the less-affected body side showed a PA distribution similar to free-living, including vigorous intensity. For the lower body, similar distributions can be observed for affected and less-affected side in free-living and during therapy. We attributed the similarity in lower body sides to bi-pedal locomotion and postures including body sides equally. Lower body PA differences suggested subtle compensation mechanisms compared to the upper body. However, the majority of PA was categorized as light to moderate.

### 3.2. Physical activity and motion intensity

**Figure 6:**
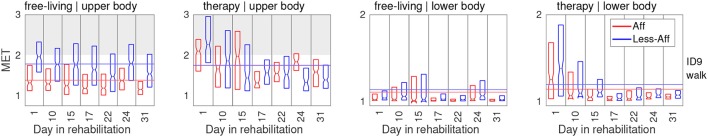
PA in MET equivalents for affected and less-affected upper and lower body for patient ID9. To optimize readability, first and third quartile are shown. Notches represent median MET equivalent values.

To investigate intra- and inter-patient PA variation, MET equivalents were estimated for each day of the rehabilitation period and visualized sequentially as boxplots. Figure 6 illustrates the daily estimated MET distribution during the rehabilitation period for patient ID9 exemplarily. For example, PA of patient ID9 was on average 22.5 % higher in the less-affected side (1.78 MET) compared to the affected side (1.38 MET) during free-living. During therapy sessions we observed an increased average PA of 1.75 MET in the affected side of the upper body. The affected and less-affected upper body sides showed similar PA intensity during training with an average MET equivalents difference below 1 %. During therapy we observed days with higher MET equivalents on the upper body's affected side compared with the less-affected side (recording days 15, 24, and 31). The lower body limbs showed similar MET equivalents on affected and less-affected sides with differences of 2.6 % (free-living) and 4.5 % (therapy). Illustrations of daily differences between body sides derived in free-living and therapy during the recording period of all patients are included in the Supplementary Material, see Figures S1, S2.

Table 2 summarizes estimated average MET equivalents for each patient during the complete rehabilitation duration. For each patient, except ID4, we observed higher or similar MET equivalents in the upper body's affected side during therapy, compared to free-living. For the less-affected upper body side, eight patients including ID1–4, and ID7–10, showed higher or similar MET equivalents during free-living compared to therapy. For the lower body, average values for each patient were similar between affected and less-affected sides during free-living and therapy. Mean values of upper body's affected side indicated that patients benefit from therapy session due to increased MET equivalents compared to free-living. The upper body's less-affected side, and the lower body showed similar average MET equivalents across all patients, independent of the rehabilitation setting. Table 2 includes the PA comparison between affected and less-affected body sides in free-living and therapy. In free-living, PA was on average 28.4 % higher in the less-affected arm compared to the affected arm. In therapy, PA was on average 15.4 % higher in the less-affected arm compared to the affected arm. Further, PA of the affected arm was on average 16.1 % higher during therapy compared to free-living. In contrast, PA of the affected leg was on average only 5.3 % higher during therapy compared to free-living.

**Table 2:** Average MET equivalents for affected and less-affected upper and lower body sides during free-living and therapy.

	**Free-living**				**Therapy**				**Aff vs. Less-Aff**				**Free-living vs. Therapy**			
									**Difference [%]**				**Difference [%]**			
	**Aff**	**NonAff**	**Aff**	**NonAff**	**Aff**	**NonAff**	**Aff**	**NonAff**	****Free-living****	****Free-living****	**Therapy**	**Therapy**	**Aff**	**NonAff**	**Aff**	**NonAff**
**ID**	**Upper**	**Upper**	**Lower**	**Lower**	**Upper**	**Upper**	**Lower**	**Lower**	**Upper**	**Lower**	**Upper**	**Lower**	**Upper**	**Upper**	**Lower**	**Lower**
1	1.14	1.64	1.04	1.08	1.41	1.53	1.10	1.15	30.6	4.3	7.9	4.1	19.3	−7.1	6.2	6.0
2	1.19	1.59	1.09	1.14	1.23	1.47	1.09	1.13	24.8	4.3	16.5	3.2	2.9	−7.8	0.4	−0.7
3	1.16	2.10	1.01	1.06	1.36	1.86	1.15	1.13	44.7	4.7	27.1	−2.0	14.3	−13.1	12.4	6.3
4	1.33	1.24	1.05	1.05	1.03	1.16	1.01	1.01	−7.0	0.0	11.8	0.0	−29.3	−6.6	−4.3	−4.3
5	1.76	2.17	1.08	1.10	2.51	2.40	1.17	1.22	19.2	1.8	−4.6	3.9	30.1	9.5	7.8	9.8
6	1.13	1.47	1.10	1.11	1.34	1.58	1.13	1.13	23.2	0.7	15.0	−0.6	15.7	6.7	2.9	1.6
7	1.16	2.42	1.05	1.09	1.47	2.07	1.13	1.13	52.2	3.3	28.7	0.0	21.5	−17.0	6.5	3.4
8	1.30	1.53	1.09	1.10	1.42	1.47	1.12	1.13	14.8	1.3	3.2	0.3	8.2	−4.3	3.1	2.1
9	1.38	1.78	1.11	1.14	1.75	1.75	1.14	1.20	22.5	2.6	0.1	4.5	21.0	−1.8	3.0	4.9
10	1.29	3.62	1.08	1.11	1.84	2.98	1.18	1.21	64.3	2.9	38.5	2.9	29.5	−21.4	8.6	8.6
11	1.87	2.42	1.13	1.14	3.32	4.46	1.28	1.30	22.7	0.5	25.7	0.9	43.5	45.7	11.9	12.3
Mean	1.34	2.00	1.07	1.10	1.70	2.07	1.14	1.16	28.4	2.4	15.4	1.6	16.1	−1.6	5.3	4.5
SD	0.25	0.67	0.04	0.03	0.67	0.94	0.07	0.07	19.4	1.7	13.4	2.2	18.7	18.2	5.0	4.8

Average differences in MET equivalents were compared between affected and less-affected body sides during free-living and therapy in columns marked with “Aff vs. NonAff.” In addition, differences in MET equivalents were analyzed for each limb during free-living and therapy in columns marked with “Free-living vs. Therapy.”

### 3.3. Statistical analysis

Statistical analysis included significance evaluation and measuring effect size. Average differences between body sides and body parts are summarized in Table 3. All patients showed statistical significant differences in upper body MET equivalents when comparing affected and less-affected sides during free-living (Rank-sum test, *p* < 0.05). All patients, except ID4 showed higher PA intensity in the affected side. Further, all patients (except ID6 and ID11, both walkers) showed significant differences between body sides during free-living in the lower body too. For all patients, the PA intensity was higher or equal in the less-affected side compared to the affected side.

**Table 3:** Statistical analysis of differences between affected and less-affected body sides for MET equivalents during free-living and therapy.

	**Free-living**				**Therapy**			
	**Rank-sum test [h]**	**Rank-sum test [h]**	**MESU3**	**MESU3**	**Rank-sum test [h]**	**Rank-sum test [h]**	**MESU3**	**MESU3**
**ID**	**Upper body**	**Lower body**	**Upper body**	**Lower body**	**Upper body**	**Lower body**	**Upper body**	**Lower body**
1	1**	1**	0.90	0.75	1*	1**	0.51	0.60
2	1**	1**	0.83	0.69	1**	1**	0.76	0.62
3	1**	1**	0.98	0.80	1**	1**	0.78	0.50
4	1**	1**	0.45	0.53	1**	1**	0.61	0.60
5	1**	1**	0.70	0.62	0	1*	0.53	0.54
6	1**	0	0.78	0.54	1**	1*	0.65	0.47
7	1**	1**	0.99	0.69	1**	0	0.81	0.50
8	1**	1**	0.68	0.60	0	0	0.51	0.50
9	1**	1**	0.76	0.64	0	1**	0.45	0.62
10	1**	1**	1.00	0.66	1**	1**	0.81	0.54
11	1**	0	0.64	0.49	1**	0	0.58	0.55
Mean			0.79	0.64			0.64	0.55
SD			0.17	0.10			0.13	0.06

The Rank-sum test evaluates the null hypothesis (**H**_0_) that MET equivalents of affected and less-affected body sides have equal medians, against the alternative hypothesis (**H**_1_) that they have not, using an arbitrary α-value. Hence, the Rank-sum test results in a rejection or acceptance of **H**_0_ and is marked with 1 and 0, respectively. Significance analysis results for p < 1 % are marked with **, for p < 5 % marked with *. Measure of effect size (MESU3) values range from 0 to 1. A MESU3 value of 0.5 indicates that there is no effect between affected and less-affected sides.

MESU3 during free-living supported the Rank-sum test decision of significance for the upper and lower body in 10 patients. For patient ID4, MESU3 resulted in 0.45 (upper body) and 0.53 (lower body) during free-living, indicating no difference between body sides, contrary to significant test decisions derived by the Rank-sum test. Average MESU3 values further confirmed that differences between body sides were higher in free-living (0.79 ± 0.17 for affected side; 0.64 ± 0.1 for less-affected side) compared to differences found during therapy (0.64 ± 0.13 for affected side; 0.55 ± 0.06 for less-affected side). During therapy when the affected body side was trained, measures of effect size remained close to the neutral value of 0.5. Neutral MESU3 values contradict significant test results, i.e., for upper body (ID1) and lower body (ID3, ID5, ID6, and ID10).

## 4. Discussion

### 4.1. Physical activity

We investigated PA using MET equivalents to obtain insights in patient activity during a longitudinal rehabilitation process over several weeks. In particular, we compared affected and less-affected body sides and body parts during free-living and therapy. In general, PA was higher or similar in the less-affected side compared to the affected side. By distinguishing free-living and clinician guided therapy we created two distinct rehabilitation settings for this PA analysis. The free-living setting including unguided ADL, relates to a natural environment, patients will likely face after the clinical rehabilitation. In contrast, fitness training in the gym and vibration training were considered unlikely in patients' daily life following the clinical rehabilitation. Moreover, vibration training, which was not part of the standard rehabilitation programme, would not add valuable insights on individual limbs and differences due to the passive vibration of the complete body. Also, the PA analysis of unguided fitness training was not considered beneficial to investigate patients progress and potential recovery trends. Consequently, vibration and fitness training was excluded.

Our results are in line with previous published study results, though, activity intensity of limbs was typically quantified as duration of use or non-use using a threshold. For example, Michielsen et al. (2012) quantified the duration of upper limb use in chronic stroke outpatients during a 24 h recording period using accelerometers. Results showed considerable non-use of the affected side compared to the less-affected side. A threshold was fitted to the acceleration signal to distinguish use and no-use. Similarly, Lang et al. (2007) investigated affected and less-affected upper limbs using wrist-worn uni-axial accelerometers during a 24 h assessment and found that patients with hemiparesis used both body sides substantially less than healthy controls. The regression-based approach presented in this work was derived to evaluate PA on a continuous scale using MET equivalents, hence subtle changes and differences between body sides can be quantified.

As expected, patients included in this study, showed often higher intensity in the upper body's affected side during therapy compared to free-living. Higher PA during therapy could indicate patients' movement potential, thus help devising personalized exercise recommendations and target planing to increase PA of the affected side. Based on the analysis between affected and less-affected arms, Bailey et al. (2015), suggested that increasing activity of the less-affected side might increase the activity of the affected side due to the bilateral nature of everyday tasks. Beside therapy, which trains the affected side, exercises during free-living, including bi-manual tasks could further promote recovery in the affected side.

Our analysis showed differences in PA between therapy and free-living independent of age, EBI scores, and days since stroke.

Variances in patient therapies and individual behavior suggested that personalized therapy strategies could best fit patient-specific needs, hence maximize recovery outcome. Results from this PA analysis align with our previous work, demonstrating that particularly walking behavior was influenced by the therapy schedule, patient-individual habits, and patients' daily health condition rather then patient-specific characteristics, rendering generalization difficult (Derungs et al., 2018a). In addition, we estimated increased range of motion in the affected arm during clinician guided therapy compared to free-living (Derungs et al., 2018b). In contrast, the bi-pedal locomotion of walkers and reduced leg movement in wheelchair users, led to similar leg PA in free-living and therapy.

Thresholds to separate distinct intensities vary across different published studies, thus comparing absolute MET equivalent levels derived in this work with related work appeared inappropriate. A tendency toward light PA was evident in the present study. Our observation relates well to previous studies describing sedentary behavior among patients after stroke. Paul et al. (2016) showed that sedentary time was higher in patients after stroke compared to healthy controls, measured for seven consecutive days using an accelerometer-based wearable sensor. Moreover, Moore et al. (2013) showed that PA reduced immediately after the stroke and remained below recommended levels. Sedentary behavior was observed up to six months post-stroke. Our approach is sensitive to estimate PA in individual limbs to indicate sedentary behavior in free-living and therapy.

### 4.2. Physical activity—is there a recovery trend?

So far, clinical assessment scores to describe the recovery progress were estimated using classification or regression methods (Derungs and Amft, 2018). However, the scores considered specific motor tasks only and generalization to free-living remains unclear. Buma et al. (2013) emphasized that the first six month are crucial for upper limb recovery and skill-learning. Hence, measurements ranging from 24 h to one week (Moore et al., 2013; Bailey et al., 2015) seem insufficient. In previous work, we demonstrated that the EBI score of this study population could be estimated without specific tests considering distinct activity primitives (Derungs et al., 2015). In this analysis we qualitatively analyzed if increasing PA intensity in individual limbs could indicate a recovery trend during the rehabilitation. We found recording days, where patients showed higher PA intensity in the affected side compared to the less-affected side. However, PA varied daily in body sides and body parts across all patients due to individualized therapy, and patients' condition. A clear recovery trend with increasing PA was not found. Contrary, our results confirmed a tendency to sedentary behavior in patients after stroke, independent of free-living or therapy.

### 4.3. Statistical analysis

Verschuren et al. (2015) described MET equivalent values below 1.5 MET as sedentary behavior. However, definitions for separating intensity levels in patients after stroke vary. Hence, we analyzed PA differences between body sides, avoiding comparing absolute MET equivalent values. The non-parametric Mann-Whitney *U*-test was suitable for non-normal distributed data. However, the binary significance test is not sufficient to measure subtle changes during the rehabilitation, even when considering *p*-values. On the other hand does a non-significant test result not imply an ineffective rehabilitation because small sample size and measurement variability may affect the statistical result (Page, 2014). Our analysis included the MESU3 to measure the magnitude of differences between affected and less-affected sides. MESU3 supported mostly test decisions derived using the Mann–Whitney *U*-test. Hence, assessing PA differences during stroke rehabilitation should include the MESU3, to quantify subtle changes on a continuous scale.

### 4.4. Clinical implications and limitations

Long-term monitoring and trend analysis of patients after stroke in rehabilitation and free-living is challenging. For example, validating MET estimation algorithms require lab-controlled settings, reference systems and scripted activities (Staudenmayer et al., 2015). In particular, acquiring ground truth of patients' activities limits free-living validation. Validation including breath-by-breath spirometry is similarly infeasible due to the high patient burden. In contrast, regression methods based on acceleration measurements are independent of data-based training (Ohkawara et al., 2011; Mortazavi et al., 2013). Our results provided insights on the differences in activity patterns of all extremities.

Although we defined inclusion criteria to analyse PA in a homogeneous study population, the influence of impairments and other factors, e.g., age, days after stroke, EBI scores, mood, pain, etc., were not monitored in this observation study. However, previous analysis, including the same study population, suggested that patient-specific therapy schedules and therapy programmes influenced the patient's upper extremity range of motion (Derungs et al., 2018b) and mobility behavior (Derungs et al., 2018a) independent of age, days after stroke or EBI scores.

In modern stroke rehabilitation, exercises and therapy is adapted to each patient's needs and health conditions. Consequently, assessing PA is challenging, in particular in remote monitoring. Nevertheless, increasing PA is essential to avoid subsequent strokes and co-morbidities, e.g., cardiovascular diseases might be reduced when adapting rehabilitation exercises to higher PA intensities (Usui and Nishida, 2015). Billinger et al. (2014) suggested peak exercise training including intensities up to 5 MET equivalents. Exercise recommendations for patients after a myocardial infarction could be integrated in stroke rehabilitation and controlled continuously using PA feedback derived from wearable sensors.

External motivation, i.e., gaming exercises (Kafri et al., 2014) or virtual reality (Subramanian et al., 2013), should be integrated into the daily rehabilitation programme. Gaming and continuous sensor-based PA evaluation could be used as supportive rehabilitation tool. For example, sedentary patients could play balance games where standing postures are required, bi-manual ADL tasks should be encouraged in free-living. However, patients might be motivated during the clinical rehabilitation, transferring approaches to free-living for continuous PA quantification are required. Motivation methods that could promote PA and thus recovery should include objective, sensor-based evaluation of understandable health recommendations (Baert et al., 2012).

The population size was limited in this study. Our approach including wearable motion sensors could facilitate PA monitoring in larger study populations to investigate how PA is influenced across different levels of impairments (Kollen et al., 2006). Further, unobtrusive, textile-integrated sensors, e.g., as described by Harms et al. (2009), could be helpful for PA monitoring of hemiparetic patients, as they may not be able to handle sensors separately.

## 5. Conclusion

We investigated PA intensity of eleven hemiparetic patients during a longitudinal outpatient rehabilitation period of several weeks. We compared affected and less-affected body sides and body parts during a standard rehabilitation programme in a day-care center, including free-living and clinician guided therapy settings. In this analysis, we focused on PA of each limb using wrist- and thigh-worn sensors. PA intensity was estimated in MET equivalents rendering interpretation comparable, hence patients' performance can be assessed quickly by clinicians.

We demonstrated that wearable motion sensors provide insights in patients' PA intensity during rehabilitation. In particular, we showed that PA during unguided ADL tasks in free-living differs from therapy settings, where patients follow clinician guided exercises. We confirmed previous published lab-study results indicating that sedentary behavior prevails in patients after stroke. No recovery trend was observed. The differences between free-living and therapy were observed although special, non-standard training sessions were excluded. Results showed on average higher PA intensity during therapy compared to free-living; 16.1 % in the affected arm and 5.3 % in the affected leg. Differences between therapy and free-living were below 4.5 % in the less-affected arm and leg.

We expect that wearable sensors could enable longitudinal, remote PA evaluation of each limb beyond clinical rehabilitation settings to quantify intensity and devise personalized exercise recommendations for free-living.

## Ethics statement

Following detailed verbal and written information about the character of the study, all participants signed an informed consent according the declaration of Helsinki before the recording began. Participants further approved the publication of patient details and study results according the signed informed consent. The protocol of the study was approved by the Swiss cantonal Ethics committee of the canton Aargau, Switzerland (Application number: 2013/009).

## Data availability

As declared and approved in the Ethics application, all data remain sole property of involved research institutions, namely: ACTLab (Friedrich-Alexander Universität Erlangen-Nürnberg, TU Eindhoven), IfE (ETH Zürich), and Reha Rheinfelden. Following the Reha Rheinfelden policy, the data repository remains confidential.

## Author contributions

AD data recordings, algorithm development, analysis. CS-A supported the study design, Ethics application and approval process, helped with patient recruitment and inclusion. OA helped in all facets of the study and manuscript preparation. All authors read and approved the final manuscript.

### Conflict of interest statement

The authors declare that the research was conducted in the absence of any commercial or financial relationships that could be construed as a potential conflict of interest.

## Footnotes

^1^Shimmer 9DoF Calibration Application User Manual Rev. 2.2a, 2013.

^2^MATLAB, Release 2013b, The MathWorks, Inc., Natick, MA, United States.

^3^Average of Swiss gravity zones according https://www.metas.ch/metas/de/home/dok/gravitationszonen.html
