# Supplementary material for: Physical Activity Comparison Between Body Sides in Hemiparetic Patients Using Wearable Motion Sensors in Free-Living and Therapy: A Case Series
Source: Front Bioeng Biotechnol. 2018 Oct 17;6:136. doi: 10.3389/fbioe.2018.00136 (PMC6199363; doi:10.3389/fbioe.2018.00136)
Supplement: Supplementary file 1 [file Data_Sheet_1.pdf]

Supplementary Material:
Physical Activity Comparison between Body sides in Hemiparetic Patients using Wearable Motion Sensors in Free-living and Therapy: A Case Series

The MET equivalents of patients ID1-ID5 and ID6-ID11 are illustrated in Figures S1 and S2, respectively.

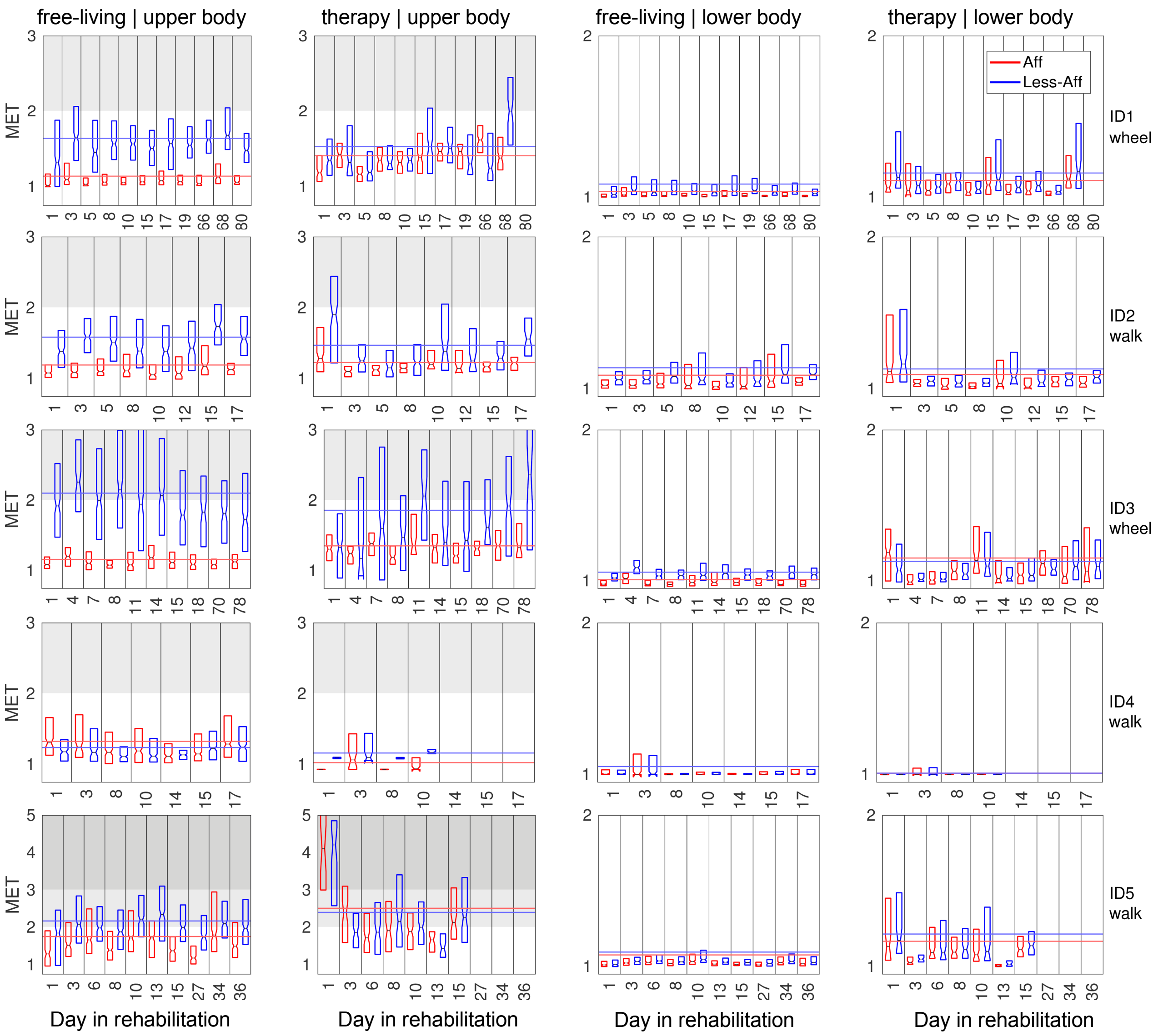

Figure S1. PA in MET equivalents for affected and less-affected upper and lower body. The plots show daily PA intensity estimates of patients ID1 to ID5 during the rehabilitation period. Walking patients are denoted with ’walk’; wheelchair users are marked with ’wheel’. To optimize readability, first and third quartile are shown. Notches represent median MET equivalent values.

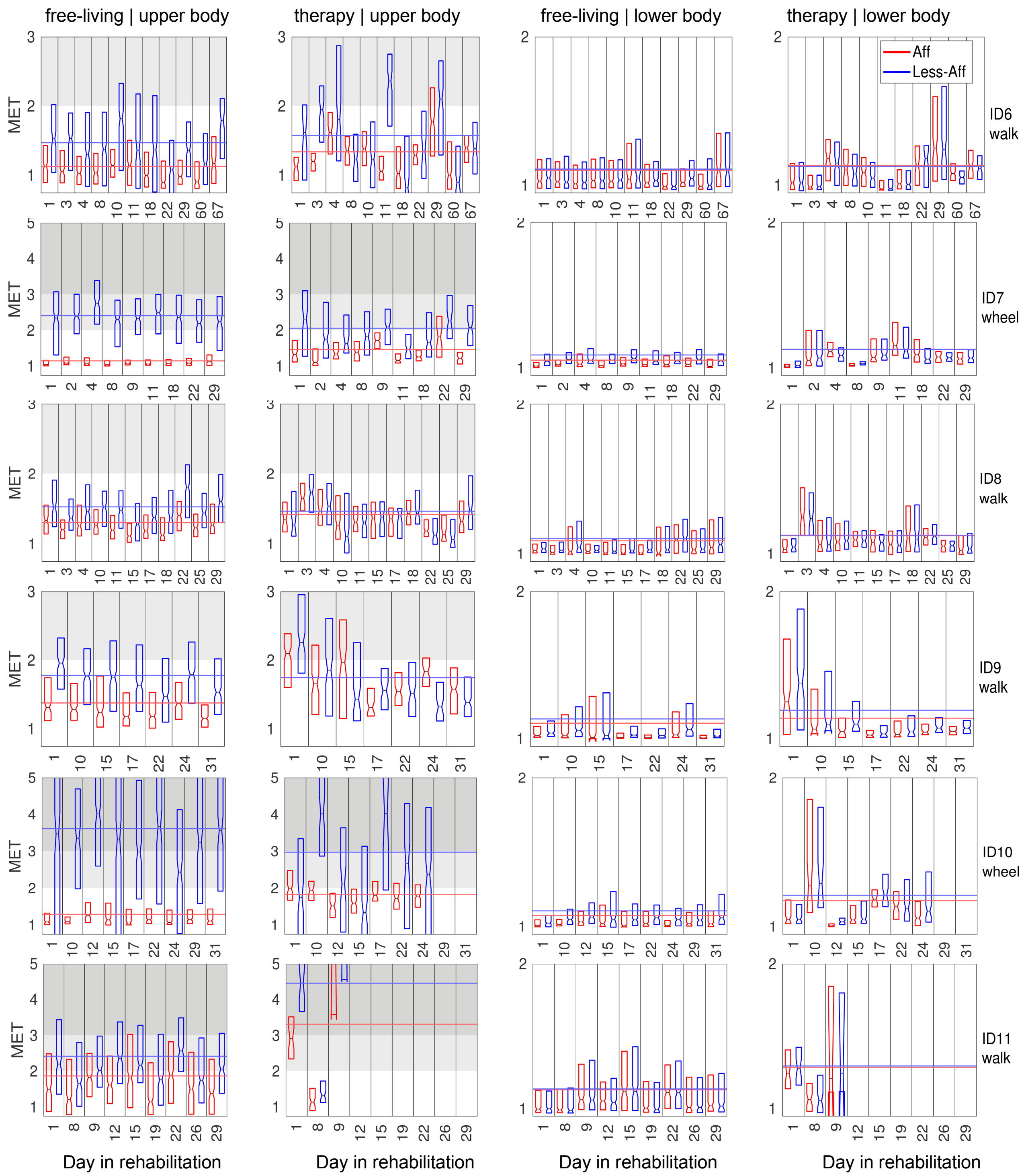

Figure S2. PA in MET equivalents for affected and less-affected upper and lower body. The plots show daily PA intensity estimates of patients ID6 to ID11 during the rehabilitation period. Walking patients are denoted with 'walk'; wheelchair users are marked with 'wheel'. To optimize readability, first and third quartile are shown. Notches represent median MET equivalent values.
